# Dietary Effect of Palm Kernel Oil Inclusion in Feeding Finishing Lambs on Meat Quality

**DOI:** 10.3390/ani12233242

**Published:** 2022-11-23

**Authors:** Daniela Pionorio Vilaronga Castro, Paulo Roberto Silveira Pimentel, Neiri Jean Alves dos Santos, Jarbas Miguel da Silva Júnior, Gercino Ferreira Virginio Júnior, Ederson Américo de Andrade, Analívia Martins Barbosa, Elzânia Sales Pereira, Cláudio Vaz Di Mambro Ribeiro, Leilson Rocha Bezerra, Ronaldo Lopes Oliveira

**Affiliations:** 1 Department of Animal Science, Federal University of Bahia, Salvador 40170-110, BA, Brazil; 2 Department of Animal Science, Federal University of Ceara, Fortaleza 60021-970, CE, Brazil; 3 Department of Animal Science, Federal University of Campina Grande, Patos 58708-110, PB, Brazil

**Keywords:** CLA, dodecanoic acid, lamb meat

## Abstract

**Simple Summary:**

Palm kernel oil (PKO) is extracted from an oleaginous seed fruit (*Elaeis guineenses* Jacq.), commonly cultivated in Brazil, and it can be used strategically as a ruminal fermentation modulator and possibly affect the meat quality. We conducted an experimental trial increasing PKO levels in the diet of lambs, and we evaluated different meat quality parameters, such as carcass weight and characteristics, the yield of commercial cuts, physicochemical composition, fatty acid profile, and sensorial consumer perception. However, we observe that palm kernel oil in the diet of lambs up to the level of 5.2% did not change the physicochemical attributes of lamb meat. However, it reduced the slaughter body weight and carcass traits weight and yield, C18:3 and n-3 FA content, and juiciness by sensory panel perception, thus from the point of view of lamb meat production and fatty acid profile, the inclusion of PKO is not beneficial.

**Abstract:**

This study evaluated the effects of palm kernel oil (PKO) in the diet of lambs on carcass characteristics, quality, and fatty acid profile of the meat. Forty uncastrated male Santa Inês lambs were used and divided among the treatments: PKO_zero_ without inclusion; PKO_1.3_—added 1.3%; PKO_2.6_—added 2.6%; PKO_3.9_—added 3.9%; PKO_5.2_—added 5.2%. The carcass characteristics, the variables related to meat color, and the chemical composition of the *Longissimus lumborum* of lambs were not affected by the PKO inclusion. The weight of the carcasses at slaughter, hot and cold, half carcass, loin-eye area, and commercial cuts decreased linearly when PKO was added to the lamb diet (*p* < 0.01). CCY decreased linearly to the inclusion level of 2.66% PKO (RMSE 2.204). Total conjugated linoleic acid CLA and C18:3 n-3 GA concentrations remained stable until the inclusion levels of 3.44% PKO (RMSE 0.0956) and 2.17% (RMSE 0.0637), decreasing its concentrations as the increased level of PKO. The presence of PKO in the lambs’ diet up to the level of 5.2% did not change the meat quality characteristics; thus, from the point of view of lamb meat production and fatty acid profile, the inclusion of PKO is not beneficial.

## 1. Introduction

Customers are more demanding, looking for nutritional foods beneficial to health [1]. Thus, functional foods can be defined as foods containing biologically active substances in their composition that stimulate positive metabolic effects in the organism [2]. Many biological effects are associated with bioactive compounds, such as the reduction of neurodegenerative diseases (Parkinson’s and Alzheimer’s disease) and chronic diseases (cardiovascular diseases, diabetes and several types of cancer) [1,3]. The bioactive compounds can be either of plant origin, such as phytonutrients such as carotenoids and phytosterols, or animal origin, such as the essential fatty acids (FA) present in fish oil and conjugated linoleic acid (CLA) present in meat products from ruminants [1,2,3,4].

The CLA corresponds to a mixture of geometric isomers of linoleic acid (C18:2n-6), being products of the incomplete biohydrogenation process of this FA in the rumen [2]. Among the main CLA, C18:2 *cis-*9 *trans-*11 and C18:2 *trans-*10 *cis-*12 acids stand out as the main isomers [2,5]. Several studies have used vegetable oils due to their effects on the ruminal biohydrogenation process to increase the concentration of this compound, especially in meat [6,7,8]; mainly, those oils rich in medium-chain FA can be used for their effect on the stability of rumen microorganisms [6,9,10,11].

Palm kernel oil (PKO) is the product of processing the seeds of the palm fruit (*Elaeis guineensis* Jacq.), and it is rich in medium-chain FA such as lauric and myristic acids [12,13]. Lauric acid affects microorganisms and the de novo synthesis of triacylglycerols, changing its amount in the cell membrane, modifying its fluidity and permeability, interfering in metabolism, and controlling the entry and exit of substances [14,15,16]. Therefore, the presence of PKO as a source of lauric acid can affect the ruminal bacterial metabolism and consequently modify ruminal biohydrogenation, altering the profile of fatty acids incorporated in meat.

The use of palm kernel cake and oil, byproducts of palm fruit production, can have economic benefits in ruminant feed [17,18], maintaining physicochemical, sensory and meat acceptance characteristics [17,19]. However, it is not known whether palm kernel oil in lamb diets can affect meat quality.

Thus, this study aims to evaluate the effect of increasing levels of palm kernel oil (up to 5.2%) in the diet and its effects on meat quality. We hypothesize that the inclusion of palm kernel oil, as a source of lauric acid, promotes an increase in carcass weight and modifies the fatty acid profile of meat with increasing concentrations of unsaturated fatty acids CLA and n-3 and n-6.

## 2. Materials and Methods

### 2.1. Animals, Diets, Management, and Experimental Design

The study used 40 uncastrated male Santa Inês lambs, with initial body weight (BW) of 25.73 ± 4.06 kg (mean ± standard deviation, respectively). The animals were previously identified, vaccinated, dewormed, and housed in individual suspended stalls (1 m^2^) with wooden slatted floors. The animals were randomly distributed into five palm kernel oil inclusion levels in the diet, totaling 8 animals per treatment. The treatments consisted of the inclusion of PKO (PalmFry, Jundiaí, Brazil) in the diet (Supplementary Table S1), as follows: PKO_zero_—no inclusion of palm kernel oil; PKO_1.3_—added 1.3% of palm kernel oil; PKO_2.6_—added 2.6% of palm kernel oil; PKO_3.9_—added 3.9% of palm kernel oil; PKO_5.2_—added 5.2% of palm kernel oil, based on dry matter of the total diet.

The animals were fed ad libitum, but there were two feeding times (08:00 and 16:00 h), with total mixed ration (TMR) and free access to water. Daily, the leftovers were collected from the trough and weighed, and the offered meals were then adjusted based on leftovers compared with the offered amount, allowing for 10–20% leftovers. The diets were formulated according to the National Research Council [20] to provide an average daily gain (ADG) of 200 g (Table 1), with a roughage: concentrate ratio of 40/60. The concentrate was formulated with ground corn, soybean meal, mineral mixture and PKO. As roughage, Tifton-85 hay (*Cynodon* sp.) ground in a particle of approximately 5.0 cm was used. Performance evaluation lasted 96 days, with 15 days of adaptation to the environment, management, and diets.

### 2.2. Slaughter, Commercial Cuts, Meat Sampling, and Chemical Composition

Performance evaluation lasted 96 days, with 15 days of adaptation to the environment, management, and diets. The animals were weighed at the beginning and every 25 days to follow the ADG. At the end of the experimental period, the animals were weighed after a 16 h fast of solids and a hydric diet to obtain the slaughter body weight (SBW). Then, the animals were transported to a commercial slaughterhouse located approximately 20 km away, as the travel time was less than 45 min. The slaughter was performed following the guidelines for humane slaughter as stated by the Federal Inspection Service, in accordance with regulations (Brasil [21], Normative n. 03/00, Agriculture and Livestock). The animals were stunned by electronarcosis, followed by bleeding, skinning, evisceration, and removal of the head and limbs.

After removing the organs, feet, head, and skin, the carcasses were weighed to obtain the hot carcass weight (HCW) and then stored for 24 h in a cold chamber (4 °C), being weighed again to obtain the cold carcass weight (CCW). The commercial carcass yield (CCY) was determined according to the following calculation:

CCY (kg) = (CCW/SBW) × 100(1)

After cooling (4 °C for 24 h), the carcasses were longitudinally sectioned, and the left half of the carcass was divided into five anatomical regions, considered as commercial cuts, being: neck (1st to 7th cervical vertebra), shoulder (bone base scapula, humerus, and carpus), ribs (1st to 13th thoracic vertebra), loin (*Longissimus lumborum* muscle dissected from the vertebral bones) and shank (the section between the last lumbar and first sacral vertebra), according to methodology adapted from Colomer-Rocher [22]. Subsequently, the commercial cuts were weighed to determine the weights and proportions of the cuts to the half carcass weight.

Labeled foil pouches of the *L. lumborum* muscle were transported in thermoses to the Multifunctional Laboratory of the Federal University of Bahia—UFBA for determination of the loin-eye area (LEA) and subcutaneous fat thickness (SFT), desiccation and sensory analysis evaluations, and determination of the qualitative characteristics of meat: color, water holding capacity (WHC), cooking weight loss (CWL), and shear force (SF).

LEA and SFT parameters were evaluated in the left loin. After exposure of the *L. lumborum* muscle, the LEA was obtained by contouring the muscle area through the transparent sheet with a marker pen, determining the area using a computer program. The SFT was measured using a digital caliper, taking measurements at three different points of the cut, considering the average of the three points as the SFT measurement.

According to Miltenburg et al. [20], the meat color parameters were obtained. Refrigerated meat samples were previously exposed to oxygen for five minutes to promote contact of myoglobin with oxygen. Then, the samples were submitted to color determination using a Minolta CR-200 colorimeter (Konica^®^ Minolta, Osaka, Japan), using the CIE L a* b* system to determine the luminosity (L*), redness (a*) and yellowness (b*) parameters, measuring in three distinct points of the muscle to obtain the exclusive representation, and the average of the three values represented the meat color parameter. Based on the values for the intensity of red and yellow, the saturation or chroma index (C*) was calculated according to the equation described by Warriss [23] and the tint angle (*^o^Hue*) according to Macdougall [24]:

(2) $$C^{*}={\left(a^{*\;2}+b^{*\;2}\right)}^{0.5}$$

(3) $${}^{\circ}Hue=\left[tangent\;arc\left(\frac{b^{*}}{a^{*}}\right)\right]$$

The WHC was determined according to Hamm’s methodology [25] with modification in the subsample weight (2.29 ± 0.09 g). The samples were weighed on an analytical balance on filter paper and then placed between acrylic plates, and a 10 kg cylindrical weight was placed above the plates for 5 min. The samples were weighed, and through the difference in values between the initial and final weight of samples, the amount of water lost was calculated.

The CWL was performed according to AMSA [26]. The samples were weighed and subsequently grilled (George Foreman Jumbo Grill GBZ6BW, Rio de Janeiro, Brazil) with a stainless-steel thermocouple (Gulterm 700; Gulton, São Paulo, Brazil) inserted in the geometric center of each sample until it reached 71 °C. After being cooled in the open air, the samples were weighed again, obtaining, by difference, the values for cooking losses.

Subsequently, the grilled meat samples were divided into three subsamples with a thickness of 1 cm^2^ to determine the SF (N/cm^2^) using a texturometer (TAXT 2 plus^®^ Stable Micro Systems, Scarsdale, NY, USA), coupled with a Warner–Bratzler cell and blade, according to the methodology described by Shackelford et al. [27].

The chemical composition of the *Longissimus lumborum* muscle was performed by analysis in the FoodScanTM (FOSS, Hillerod, Denmark), using approximately 100 g of sample ground in a multiprocessor to a homogeneous mass [28]. The parameters evaluated were moisture, crude protein, fat and mineral matter.

### 2.3. Fatty Acid Composition

FA was extracted from L. lumborum according to the methodology described by Hara and Radin [29], methylating them according to Christie [30] and Rodrigues-Ruiz et al. [31]. The methylated samples were analyzed in a gas chromatograph (Finnigan Focus model, Varian, Palo Alto, CA, USA), with flame ionization detector, capillary (CP-Sil 88, 100 m × 0.25 mm i.d. × 0.20 μm film thickness, Varian, Palo Alto, CA, USA).

Hydrogen was used at a 1.8 mL/min flow rate as a carrier gas. The initial oven temperature program was 70 °C for 4 min time, 175 °C (13 °C/min) for 27 min time, 215 °C (4 °C/min) for 9 min time, and then increased 7 °C/min until 230 °C for 5 min, for a total of 65 min. The injector temperature was 250 °C, and the detector temperature was 300 °C.

A total of 1 μL aliquot of the esterified extract was injected into the chromatograph, and the identification of the fatty acids was obtained by comparing the retention times of the methyl esters of the samples with standards previously treated and added to the equipment (Supelco TM Component FAME Mix, cat 18919 Supelco, Bellefonte, PA, USA), obtaining the percentages of the fatty acids through the software—Chromquest 4.1 (Thermo Electron, Rodano, Italy). The fatty acids were quantified by normalization of the areas of the methyl esters, and the results obtained were expressed in % FAME.

The sum (Σ) of the total saturated fatty acids (ΣSFA), monounsaturated fatty acids (ΣMUFA), PUFA (ΣPUFA) and the desirable fatty acids (ΣDFA), ΣPUFA: ΣSFA, and Σn-++++++++6: Σn-3 ratios were calculated from the FA composition. From the fatty acid profile, the atherogenicity (AI) and thrombogenicity indices (TI) were calculated [32], using the following equations:

AI = [C_12_ + (C_14_ × 4) + C_16_]/ΣMUFA (4)

(5) $$TI=\frac{C_{14:0}+C_{16:0}+C_{18:0}}{\left[\left(0.5\times \Sigma MUFA\right)+\left(0.5\times \Sigma n6\right)+\left(3\times \Sigma n3\right)+\left(\frac{\Sigma n3}{\Sigma n6}\right)\right]}$$

The fatty acids omega-6 (n-6) and omega-3 (n-3) families and the ratio between them were also calculated. The activity of the enzymes ∆9-desaturase C16, ∆9-desaturase C18, and elongase was determined according to the equation described by Raes [33]:

(6) $${∆}^{9}-desaturase\;C16=\left[\frac{\left(C16:1\;cis-9\right)}{\left(C16:1\;cis-9+C16:0\right)}\right]\times 100$$

(7) $${∆}^{9}-desaturase\;C18=\left[\frac{\left(C18:1\;cis-9\right)}{\left(C18:1\;cis-9+C18:0\right)}\right]\times 100$$

(8) $$Elongase=\left[\frac{\left(C18:0+C18:1\;cis-9\right)}{\left(C16:0+C16:1\;cis-9+C18:0+C18:1\;cis-9\right)}\right]\times 100$$

### 2.4. Sensorial Analysis

AMSA [24] recommended the effective acceptance test that evaluated the sensorial attributes and consumer preference using a hedonic scale and panel composed of 85 untrained testers. The sensorial attributes were collected in individual booths under controlled temperature and lighting conditions. A structured nine-point scale (scores ranged from 1 to 9 as follows: 1, extremely unpleasant to 9, extremely likable) was used to evaluate the sensorial characteristics, and the following attributes were evaluated: flavor and “sheep” odor, softness, juiciness, and overall acceptance [26].

Raw meat samples of *L. lumborum* from the right loin of each treatment (PKO_zero_, PKO_1.3_, PKO_2.6_, PKO_3.9_, and PKO_5.2_) were cut into cubes (approximately 2.0 cm^3^), weighed (approximately 17g), and grilled in a preheated electric oven (Tramontina^®^, 25683100, Sao Paulo, Brazil) at 170 °C until the temperature of the geometric center of the sample reached 71 °C [26]. The samples were then placed in previously identified Petri dishes covered with aluminum foil to minimize heat loss and kept in a water bath (Marconi—Piracicaba—SP, Brazil) at 75 °C to maintain the temperature of the samples between 65 and 70 °C until they were served to the tasters. No salt or any kind of condiments was added to the samples. Drinking water at room temperature and a water and salt cookie were provided to the testers to remove the residual flavor between the samples.

### 2.5. Statistical Analysis

The data obtained were analyzed using PROC MIXED of SAS 9.4 software in an entirely randomized design, using the following mathematical model:

Y_i_ = μ + T_i_ + ε_i_ (9)

where Yi is variable response, µ is the overall mean, T_i_ is the fixed effect of the treatment, and εi is the experimental error.

The data obtained regarding the sensorial analysis of the lamb loin were analyzed using PROC MIXED of SAS 9.4 software, in a randomized block design, with the taster as the block, considering the normal distribution of the variables based on the graphical evaluation of the Q-Q plot and histogram, according to the model described below:

Y_ij_ = μ + T_i_ + p_j_ + ε_ij_ (10)

where Yij is variable response, µ is the overall mean, T_i_ is the treatment effect, p_j_ represents the taster, this effect being random, and ε_ij_ as the experimental error.

The heterogeneity of variances was tested using the REPEATED command and was used when significant. The covariance structures “diagonal and variance compounds” were tested and defined according to the lowest value obtained for “Akaike’s Information Criterion corrected” (AICC). Polynomial contrasts were used to test the linear and quadratic effects of palm kernel oil supply in the lamb diet on all parameters. Initial body weight was tested as a covariate and used when significant. The taster was used as the block for the sensory analysis data, and this effect was random.

The PROC NLIN command was used for the linear plateau response analysis observed for variables related to carcass yield and loin fatty acid concentration, using as a selection criterion the lowest value of the root mean square error (RMSE) between the linear and quadratic contrasts and the linear plateau response. The significant effect was declared when *p* ≤ 0.05, considering the trend when 0.05 > *p* ≤ 0.10.

## 3. Results

SBW (*p* = 0.002), HCW (*p* < 0.001) and CCW (*p* < 0.001), as well as LEA (*p* < 0.001), decreased linearly with an increase in the level of PKO supplied in the diet (Table 2), and SFT was not affected. There was a linear decreasing effect for the variables of half carcass weights (*p* < 0.001), neck (*p* < 0.001), shoulder (*p* = 0.009), shank (*p* < 0.001), loin (*p* < 0.001) and ribs (*p* < 0.001). The CCY of shank and ribs were influenced by the presence of PKO in the diet, increasing the yield of the shank (*p* = 0.004) and decreasing the yield of ribs (*p* = 0.006). The yield of other cuts, such as neck and loin, was not affected. The yield of the shoulder cut tended to increase linearly (*p* = 0.061) as the level of PKO inclusion in the diet increased. The CCY of lambs remained stable until the inclusion level of 2.66% PKO, with a reduction in yield as there was an increase in the inclusion level of PKO up to the level of 5.2% (∀ x ≤ 2.66 Y = 43.424; ∀ x > 2.66 Y = 43.42 + 1.63 × (2.66 − X); RMSE = 2.204).

The increase in the levels of PKO inclusion in the lamb’s diet did not affect the initial pH, final pH, WHC, and did not affect the color indices of the meat such as lightness, intensities of red and yellow, the color saturation index, and the angle of color hue (Table 3). There was a linear reduction trend for CWL (*p* = 0.093) and a quadratic behavior trend for shear force (*p* = 0.065) and meat lipid content (*p* = 0.084). No effects of PKO levels were observed on the chemical composition of the *L. lumborum* muscle of lambs in its moisture, crude protein, and mineral matter contents.

The concentrations of conjugated linoleic acid (ΣCLA) and linolenic acid (C18: 3 n-3) remained unchanged until the inclusion level of 3.44% (∀ x ≤ 3.4443 Y = 0.430; ∀ x > 3.4443 Y = 0.4299 + 0.0712 × (3.44 − X); RMSE = 0.09556) and 2.17% (∀ x ≤ 2.1718 Y = 0.272; ∀ x > 2.1718 Y = 0.2722 + 0.0357 * (2.1718 − X); RMSE = 0.06372) of PKO, respectively, observing decreasing concentrations of these fatty acids as the level of PKO increased (Table 4).

There was a linear decrease for the sum of fatty acids of the omega-3 (*p* = 0.042) and a tendency to linearly decrease for omega-6 (*p* = 0.090) when the level of participation of PKO in the diet increased (Table 4). A quadratic effect trend was observed for total polyunsaturated fatty acids (∑PUFA; *p* = 0.054), as well as for the ratio of polyunsaturated to saturated fatty acids (PUFA: SFA ratio; *p* = 0.064) and elongase enzyme activity (*p* = 0.063). The ratio of omega-6 to omega-3 fatty acids increased (*p* = 0.004) as the level of PKO in the diet also increased. The PKO increasing levels on lambs’ diet did not affect the meat sensory characteristics, such as flavor, softness, sheep flavor, sheep odor, and overall acceptance (Table 5). However, the presence of PKO in the diet quadratically affected meat juiciness (*p* = 0.009).

## 4. Discussion

The carcass characteristics (SBW, HCW and CCW) and the weights and yield of commercial cuts were influenced by the linear reduction of intake [18]. A 39.5% reduction in daily dry matter intake (DMI) is observed in the comparison between PKO_zero_ and PKO_5.2_, from 1.113 to 0.673 kg, respectively [18]. Consequently, this affected the commercial carcass yield (CCY), which values reduced linearly from the inclusion level of 2.66% PKO. The intake of large amounts of vegetable oils is usually associated with a negative effect on intake, ruminal digestion and carcass yield [34]. Recently, Santos et al. [35] observed similar results with young bulls, where the inclusion of up to 34.6 g/kg DM negatively affected intake, animal performance and, consequently, carcass characteristics. Several authors explain the deleterious effect on DMI caused by the increased intake of medium-chain fatty acids and its effect on increases in selectivity, rumen antimicrobial activity and the activation of hormones that control satiety [36,37,38,39,40]. Thus, the decrease in DMI with the inclusion of PKO reduced the availability of nutrients to promote increased weight gain and muscle deposition in the carcass, causing a reduction in the weight of commercial cuts in the carcass of lambs [34].

Although the weights of the commercial cuts decreased with the PKO inclusion, the cut yields of the neck, shoulder, and shank increased. According to Siqueira et al. [41], carcass composition can be affected by factors such as sex, genetics, and feeding. In this context, as genetics (Santa Inês breed) and sex (non-castrated males) were the same, the diet was the factor that influenced the carcass composition and the proportion of commercial cuts. However, the use of different concentrations of PKO could not promote changes in the yield of the commercial cut loin to the weight of the half carcass, which may be related to the development of this anatomical region about the others. Since it presents late muscle development in the animal [42] in comparison to the others, and the animals were slaughtered at an approximate age of 10 months, it is assumed that there was not enough time to verify the effect on the yield of this commercial cut, similar to what was observed in the other cuts.

The inclusion of PKO did not affect the quality characteristics and meat composition of the lambs, and this was consequently reflected in the sensorial analysis since no differences were observed for the attributes evaluated (except juiciness). Despite the effect of PKO inclusion on meat juiciness with quadratic behavior, it was not possible to determine the level of palm kernel oil inclusion at which there was less perception of meat juiciness since this level is outside the levels evaluated in this study. Factors such as water holding capacity and intramuscular fat content can affect meat juiciness, being positively correlated [43], but these factors were not affected by the inclusion of PKO.

Conjugated linoleic acid (CLA) and linolenic acid (C18:3 n-3) were the only lamb loin fatty acids affected by the inclusion of palm kernel oil. The lipid profile directly influences fatty acid deposition in ruminant meat. Decker and Park [2] report that cattle fed oils or oilseeds rich in polyunsaturated fatty acids (usually linoleic or linolenic) increase CLA synthesis in body tissue. Likewise, it was expected that the presence of PKO, a source of lauric acid (medium chain fatty acids), would influence ruminal biohydrogenation [14,15,16], modifying the profile of fatty acids incorporated in the meat, with the possibility of increasing CLA. However, it was possible to observe that the concentrations of CLA in the meat of lambs remained stable up to the point of 3.44% inclusion of palm kernel oil and declined when the levels of inclusion were higher. This result could then be associated with the PKO fatty acid profile with a reduction of proportion polyunsaturated fatty acids (linoleic and linolenic acid) and an increase in saturated fatty acids in the diet, linearly decreasing CLA synthesis [33]. However, even with the reduction of linolenic concentration and DM intake, the increase in the ether extract caused by the inclusion of PKO promoted a higher intake of linolenic acid (1.74, 3.04, 3.51, 4.39 and 4.36 g/day of linoleic acid); therefore, it was not limiting.

Similarly, the concentrations of alpha-linolenic fatty acid (C18:3 n-3) in the loin of lambs remained stable until the point of 2.17% inclusion of palm kernel oil, with a linear reduction from this point, where this response may be related to the decrease in this source of fatty acids in the diet of animals, which decreases the amount that can escape the biohydrogenation process to be absorbed and incorporated into the animal tissue [44].

The concentrations of arachidonic acid and docosahexaenoic acid (DHA) showed a quadratic effect; however, it was impossible to determine the inclusion level of palm kernel oil where the concentration of these fatty acids was higher since they are outside the biological limits evaluated in this study. DHA is a fatty acid essential for brain growth and development in children and is also required to maintain normal brain functions in adults, having α-linolenic acid as its precursor [45,46].

The linear reduction in omega-3 fatty acids with the inclusion of PKO caused a linear increase in the ratio of omega-6 to omega-3 family fatty acids (∑n-6: ∑n-3 ratio). The n-6: n-3 ratio is highly influenced by the fatty acid composition of the diet fed to the animals. The inclusion of sources rich in n-3 in the diet increases the n-3 deposited in meat, consequently reducing the intramuscular deposition of n-6 fatty acids and the n-6:n-3 ratio [33]. Referring to our study, the increase in the n-6:n-3 ratio with the inclusion of PKO is undesirable, as it is related to diseases and inflammatory responses [47]. Maintaining a low n-6 to n-3 ratio (ideally 1:1 to 1:4) is associated with decreased inflammatory responses by decreasing the release of pro-inflammatory substances, such as the cytokine IL-6 [48].

## 5. Conclusions

The presence of palm kernel oil in the diet of lambs up to the level of 5.2% did not change the physicochemical attributes of lamb meat. However, it reduced the slaughter body weight and carcass traits weight and yield and C18:3 and omega-3 (n-3) fatty acids content. Thus, from the point of view of lamb meat production and fatty acid profile, the inclusion of PKO is not beneficial.

## Figures and Tables

**Table 1:** Ingredients proportion, chemical composition, fatty acid profile and animal performance of the experimental diets.

Item	Palm Kernel Oil Levels (% DM Total)				
	0	1.3	2.6	3.9	5.2
Ingredients (% total diet)					
Hay	40.0	40.0	40.0	40.0	40.0
Ground corn	42.5	41.0	39.6	38.1	36.6
Soybean meal	16.0	16.2	16.3	16.5	16.7
Palm kernel oil	0.0	1.30	2.60	3.90	5.19
Mineral mixture ^1^	1.50	1.50	1.50	1.50	1.50
Chemical composition (% dry matter)					
Dry matter (% fresh matter)	86.8	86.9	87.1	87.3	87.5
Organic matter	93.9	93.9	93.9	94.0	94.0
Crude ash	6.1	6.1	6.1	6.0	6.0
Crude protein	13.9	13.9	13.8	13.8	13.7
Ether extract	1.40	2.66	3.92	5.18	6.44
Neutral detergent fiber_ap_	36.6	36.5	36.4	36.3	36.2
Acid detergent fiber	17.1	17.1	17.1	17.0	17.0
Non-fibrous carbohydrates ^2^	42.0	40.1	39.8	38.7	37.6
Total digestible nutrients	67.3	71.7	76.1	79.1	76.7
Diet energy estimate					
Metabolizable energy (MJ/kg DM)	10.66	11.48	12.30	12.85	12.41
Net energy (Mcal/kg)	1.53	1.64	1.74	1.82	1.76
Fatty acid profile (% FAME)					
C 10:0 (Capric)	0.38	2.00	2.36	2.58	2.78
C 12:0 (Lauric)	3.92	28.20	33.94	36.46	38.61
C 14:0 (Myristic)	2.84	10.62	11.77	13.00	14.20
C 16:0 (Palmitic)	56.00	52.70	49.05	50.70	50.80
C 16:1 (Palmitoleic)	0.42	0.34	0.35	0.34	0.32
C 18:0 (Stearic)	9.80	9.74	9.02	7.35	9.56
C 18:1^cis-9^ (Oleic)	42.17	32.67	31.17	29.27	27.57
C 18:2^cis-9 cis-12^ (Linoleic)	54.60	33.10	31.90	28.60	24.70
C 18:3n-3 (Linolenic)	11.18	10.41	10.40	10.38	10.07
Others	6.28	9.52	9.74	11.38	11.67
Animal performance ^3^					
Dry matter intake (kg/day)	1.113	1.092	0.861	0.816	0.673
Total weight gain (kg)	14.40	16.92	12.20	11.70	13.32
Average daily gain (kg)	0.18	0.21	0.15	0.14	0.16

^1^ Assurance levels (per kilogram of active elements): 120 g of calcium, 87 g of phosphorus, 147 g of sodium, 18 g of sulfur, 590 mg of copper, 40 mg of cobalt, 20 mg of chromium, 1800 mg of iron, 80 mg of iodine, 1300 mg of manganese, 15 mg of selenium, 3800 mg of zinc, 300 mg of molybdenum, maximum 870 mg of fluoride. ^2^ NFC = 100 − NDFap – CP – EE; ^3^ Data originally published in Castro et al. [18].

**Table 2:** Carcass characteristics, weight, and yield of commercial cuts of the carcass of lambs fed diets with different levels of palm kernel oil.

Item	Palm Kernel Oil (% DM)					SEM ^1^	*p* Value ^2^	
	0	1.3	2.6	3.9	5.2		L	Q
SBW ^3^	40.0	42.0	37.6	36.4	37.1	1.09	0.002	0.867
HCW ^4^	17.6	18.4	16.1	15.0	14.7	0.43	<0.001	0.655
CCW ^5^	17.5	18.3	16.1	15.0	14.7	0.42	<0.001	0.623
CCY ^6^	43.7	43.9	42.7	41.4	39.3	0.79	<0.001	0.127
SFT ^7^	1.58	1.54	1.46	1.98	1.58	0.16	0.496	0.884
LEA ^8^	10.8	12.3	10.8	9.15	9.37	0.45	<0.001	0.126
Weight (kg)								
½ carcass	8.40	9.28	8.10	7.39	6.88	0.39	<0.001	0.125
Neck	1.71	1.85	1.71	1.45	1.43	0.07	<0.001	0.140
Shoulder	1.64	1.73	1.54	1.43	1.50	0.05	<0.001	0.852
Shank	2.46	2.61	2.32	2.18	2.20	0.08	<0.001	0.731
Loin	1.10	1.16	1.00	0.82	0.92	0.05	<0.001	0.862
Ribs	1.63	1.67	1.37	1.33	1.28	0.05	<0.001	0.711
Yield ^9^ (%)								
Neck	19.9	20.6	21.5	20.1	19.3	0.65	0.403	0.037
Shoulder	19.2	19.2	19.5	19.8	20.8	0.44	0.061	0.315
Shank	28.9	28.8	29.1	30.0	30.2	0.40	0.004	0.411
Loin	12.8	12.9	12.6	11.5	12.5	0.50	0.232	0.522
Ribs	19.1	18.6	17.3	18.5	17.1	0.38	0.036	0.277

^1^ Standard error of the mean; ^2^ significance at *p* < 0.05 and trend between *p* > 0.05 and *p* ≤ 0.10; L, linear; Q, quadratic; ^3^ slaughter body weight; ^4^ hot carcass weight; ^5^ cold carcass weight; ^6^ commercial carcass yield; ^7^ subcutaneous fat thickness; ^8^ loin-eye area; ^9^ proportion of the weights of that cut in relation to that of the half carcass.

**Table 3:** Characteristics of *Longissimus lumborum* muscle of the lambs’ fed diets with different levels of palm kernel oil.

Item	Palm Kernel Oil (% DM)					SEM ^1^	*p* Value ^2^	
	0	1.3	2.6	3.9	5.2		L	Q
Initial pH	6.94	6.92	6.96	6.94	6.96	0.05	0.740	0.966
Final pH	6.03	6.01	5.90	6.00	5.94	0.05	0.189	0.511
CWL ^3^ (%)	21.3	25.8	19.5	19.1	17.5	2.56	0.093	0.503
WHC ^4^ (%)	71.5	72.0	71.1	70.4	69.8	1.02	0.125	0.594
SF ^5^ (N)	20.49	22.36	20.40	23.73	16.28	1.86	0.240	0.065
Color parameters index								
L* (lightness)	37.0	36.3	38.8	37.7	38.5	0.97	0.149	0.894
a* (redness)	21.4	21.1	21.6	21.3	21.0	0.35	0.662	0.482
b* (yellowness)	5.28	5.40	5.86	5.20	5.15	0.33	0.655	0.242
C* (saturation)	22.0	22.1	21.6	22.1	22.1	0.39	0.930	0.633
Hue° ^6^	14.0	15.0	13.7	14.1	13.8	0.75	0.603	0.795
Centesimal composition (%)								
Moisture	70.9	71.8	70.9	71.1	72.5	0.71	0.267	0.475
Crude protein	20.4	20.6	20.2	20.2	20.5	0.25	0.659	0.856
Fat	5.75	5.02	6.74	6.38	4.66	0.61	0.676	0.084
Mineral matter	2.44	2.57	2.17	2.31	2.34	0.19	0.458	0.628

^1^ Standard error of the mean; ^2^ Significance at *p* < 0.05 and trend between *p* > 0.05 and *p* ≤ 0.10; L, linear; Q, quadratic; ^3^ cooking weight loss; ^4^ water-holding capacity; ^5^ shear force; ^6^ color hue angle.

**Table 4:** Fatty acids profile (% FAME) of *Longissimus lumborum* muscle of the lambs’ fed diets with different levels of palm kernel oil.

Item	Palm Kernel Oil (% DM)					SEM ^1^	*p* Value ^2^	
	0	1.3	2.6	3.9	5.2		L	Q
C12:0	0.12	0.38	0.35	0.15	0.29	0.09	0.754	0.383
C14:0	2.46	3.88	3.50	3.07	3.55	0.54	0.457	0.447
C16:0	23.7	23.8	23.7	24.3	24.5	0.50	0.171	0.619
C18:0	15.7	16.7	14.8	13.3	14.9	1.06	0.309	0.841
C16:1	2.56	2.47	2.50	3.00	2.62	0.18	0.269	0.865
C18:1 t11	1.69	2.02	1.90	1.60	1.64	0.26	0.163	0.164
C18:1 c9	44.5	41.2	42.0	44.3	44.1	1.67	0.659	0.23 2
C18:2 n-6	2.17	2.09	3.05	2.12	1.69	0.35	0.398	0.062
CLA ^3^	0.43	0.44	0.42	0.40	0.30	0.04	0.045	0.177
C18:3 n-3	0.26	0.28	0.27	0.19	0.17	0.03	0.019	0.261
C20:4 n-6	0.64	0.68	0.99	0.56	0.46	0.12	0.221	0.043
C20:5 n-3	0.10	0.11	0.10	0.12	0.06	0.03	0.249	0.208
C22:5 n-3	0.21	0.19	0.28	0.22	0.13	0.04	0.360	0.104
C22:6 n-3	0.04	0.05	0.06	0.05	0.02	0.01	0.304	0.037
Others ^4^	3.53	3.40	3.50	3.85	3.41	0.31	0.834	0.765
∑SFA ^5^	44.2	48.7	44.9	43.4	45.7	1.59	0.887	0.925
∑MUFA ^6^	51.9	47.9	49.8	52.7	51.3	1.63	0.470	0.316
∑PUFA ^7^	3.93	3.95	5.29	3.19	2.90	0.53	0.095	0.054
PUFA:SFA ^8^	0.09	0.08	0.12	0.09	0.06	0.01	0.326	0.064
∑n-3 ^9^	0.40	0.44	0.42	0.36	0.25	0.06	0.042	0.135
∑n-6 ^10^	2.88	2.85	3.45	2.34	2.20	0.35	0.090	0.166
n-6:n-3 ^11^	6.96	6.47	9.78	7.76	8.21	0.36	0.004	0.023
AI ^12^	0.61	0.78	0.71	0.66	0.73	0.07	0.579	0.535
TI ^13^	1.45	1.65	1.49	1.40	1.57	0.09	0.979	0.980
h:H ratio index ^14^	1.84	1.63	1.73	1.75	1.68	0.09	0.506	0.609
Δ^9^-desaturase C16	9.75	9.40	9.52	11.0	9.59	0.57	0.489	0.744
Δ^9^-desaturase C18	74.4	71.1	73.9	76.9	74.7	2.19	0.356	0.757
Elongase	69.5	68.8	68.4	67.9	69.5	0.60	0.687	0.063

^1^ Standard error of the mean; ^2^ significance at *p* < 0.05 and trend between *p* > 0.05 and *p* ≤ 0.10; L, linear; Q, quadratic; ^3^ conjugated linoleic acid; ^4^ sum of less expressive fatty acids; ^5^ sum of saturated fatty acids; ^6^ sum of monounsaturated fatty acids; ^7^ sum of polyunsaturated fatty acids; ^8^ ratio of polyunsaturated: saturated fatty acids; ^9^ sum of omega-3 family fatty acids (18:3 n-3, 20:5 n-3, 22:6 n-3, 22:5 n-3, 18:4 n-3, 20:4 n-3); ^10^ sum of omega-6 family fatty acids (18:2 n-6, 20:4 n-6, 18:3 n-6, 20:3 n-6, 20:2 n-6, 22:5 n-6, 22:4 n-6); ^11^ ratio of omega-6 to omega-3 family fatty acids; ^12^ atherogenicity index; ^13^ thrombogenicity index; ^14^ hypocholesterolemic: hypercholesterolemic fatty acids ratio.

**Table 5:** Sensorial parameters of *Longissimus lumborum* muscle of the lambs fed with diets containing different levels of palm kernel oil.

Attributes	Palm Kernel Oil (%DM)					SEM ^1^	*p* Value ^2^	
	0	1.3	2.6	3.9	5.2		L	Q
Flavor	6.89	6.51	6.38	6.68	6.45	0.18	0.101	0.152
Softness	7.33	7.24	7.23	7.11	7.24	0.16	0.462	0.497
Juiciness	7.13	6.56	6.64	6.83	6.80	0.15	0.321	0.009
Sheep flavor	5.36	5.52	5.36	5.54	5.55	0.21	0.416	0.950
Sheep odor	4.68	4.51	4.76	4.39	4.88	0.21	0.507	0.176
Overall acceptance	6.75	6.68	6.43	6.57	6.60	0.17	0.335	0.247

^1^ Standard error of the mean; ^2^ significance at *p* < 0.05 and trend between *p* > 0.05 and *p* ≤ 0.10; L, linear; Q, quadratic.

## Data Availability

Data are not publicly available due to restrictions on the research group but can be requested from the corresponding author.

## Supplementary Materials

The following are available online at https://www.mdpi.com/article/10.3390/ani12233242/s1, Table S1. Fatty acid composition of palm kernel oil.
