# Supplementary material for: Dietary Effect of Palm Kernel Oil Inclusion in Feeding Finishing Lambs on Meat Quality
Source: Animals (Basel). 2022 Nov 23;12(23):3242. doi: 10.3390/ani12233242 (PMC9736921; doi:10.3390/ani12233242)
Supplement: Supplementary file 1 [file animals-12-03242-s001.zip › animals-1603532-supplementary.pdf]

animals

Article

Dietary Effect of Palm Kernel Oil Inclusion in Feeding Finishing Lambs on Meat Quality

Daniela Pionorio Vilaronga Castro [1], Paulo Roberto Silveira Pimentel [1], Neiri Jean Alves dos Santos [1], Jarbas Miguel da Silva Júnior [1], Gercino Ferreira Virginio Júnior [1], Ederson Américo de Andrade [1], Analívia Martins Barbosa [1], Elzânia Sales Pereira [2], Claudio Vaz Di Mambro Ribeiro [1], Leilson Rocha Bezerra [3] and Ronaldo Lopes Oliveira [1,*]

[1] Department of Animal Science, Federal University of Bahia, Salvador 40170110, BA, Brazil
[2] Department of Animal Science, Federal University of Ceara, Fortaleza 60021-970, CE, Brazil
[3] Department of Animal Science, Federal University of Campina Grande, Patos 58708110, PB, Brazil
* Correspondence: ronaldooliveira@ufba.br

Citation: Castro, D.P.V.; Pimentel, P.R.S.; dos Santos, N.J.A.; da Silva Júnior, J.M.; Virginio Júnior, G.F.; de Andrade, E.A.; Barbosa, A.M.; Pereira, E.S.; Ribeiro, C.V.D.M.; Bezerra, L.R.; et al. Dietary Effect of Palm Kernel Oil Inclusion in Feeding Finishing Lambs on Meat Quality. *Animals* **2022**, *12*, 3242. https://doi.org/10.3390/ani12233242

Academic Editors: Aser García-Rodríguez and Idoia Goiri

Received: 2 February 2022
Accepted: 16 March 2022
Published: 23 November 2022

Publisher's Note: MDPI stays neutral with regard to jurisdictional claims in published maps and institutional affiliations.

Copyright: © 2022 by the authors. Licensee MDPI, Basel, Switzerland. This article is an open access article distributed under the terms and conditions of the Creative Commons Attribution (CC BY) license (https://creativecommons.org/licenses/by/4.0/).

Table S1. Fatty acid composition of palm kernel oil.

Fatty acid	Systematic Name	Common Name	g/100g
C4:0 to C10:0	-	-	7.78
C12:0	Dodecanoic	Lauric	46.63
C14:0	Tetradecanoic	Myristic	16.05
C16:0	Hexadecanoic	Palmitic	8.59
C18:0	Octadecanoic	Stearic	2.34
C18:1^{cis9}	Octadecaenoic	Oleic	14.06
Others	-	-	4.53
Total	-	-	100.0
